# Ni-doped TiO_2_ nanotubes for wide-range hydrogen sensing

**DOI:** 10.1186/1556-276X-9-118

**Published:** 2014-03-13

**Authors:** Zhaohui Li, Dongyan Ding, Qiang Liu, Congqin Ning, Xuewu Wang

**Affiliations:** 1 Institute of Microelectronic Materials and Technology, School of Materials Science and Engineering, Shanghai Jiao Tong University, Shanghai 200240, China; 2 State Key Laboratory of High Performance Ceramics and Superfine Microstructure, Shanghai Institute of Ceramics, Chinese Academy of Sciences, Shanghai 200050, China; 3 School of Information Science and Engineering, East China University of Science and Technology, Shanghai 200237, China

**Keywords:** TiO_2_ Nanotubes, Ni doping, Hydrogen sensor, First-principles calculations

## Abstract

Doping of titania nanotubes is one of the efficient way to obtain improved physical and chemical properties. Through electrochemical anodization and annealing treatment, Ni-doped TiO_2_ nanotube arrays were fabricated and their hydrogen sensing performance was investigated. The nanotube sensor demonstrated a good sensitivity for wide-range detection of both dilute and high-concentration hydrogen atmospheres ranging from 50 ppm to 2% H_2_. A temperature-dependent sensing from 25°C to 200°C was also found. Based on the experimental measurements and first-principles calculations, the electronic structure and hydrogen sensing properties of the Ni-doped TiO_2_ with an anatase structure were also investigated. It reveals that Ni substitution of the Ti sites could induce significant inversion of the conductivity type and effective reduction of the bandgap of anatase oxide. The calculations also reveal that the resistance change for Ni-doped anatase TiO_2_ with/without hydrogen absorption was closely related to the bandgap especially the Ni-induced impurity level.

## Background

There is a strong need to develop robust hydrogen sensors for use in hydrogen cars, chemical production, and spacecraft fuel cells as well as other long-term applications [1,2]. A key requirement for these sensors is the ability to selectively detect hydrogen at lower temperatures with minimal power use and weight. Due to nanostructure-enhanced sensing capability, metal oxide nanotubes have played an increasingly important role in the last few years as gas sensing materials. Oxide nanotube has become a potential candidate for the development of the targeted robust hydrogen sensors [3-5].

TiO_2_-based gas sensors have been widely used mainly because of their inert surface properties and the change of electrical resistance after adsorption of hydrogen [6]. As a wide bandgap semiconductor material [7,8], anatase TiO_2_ (Eg ≈ 3.2 eV) usually suffers from a poor electrical conductivity and resistance increase of electronic devices; therefore, anatase TiO_2_ oxide seems to be probably hard to become an ideal material used for hydrogen detecting. However, existing literatures have demonstrated that the above problem can be effectively addressed through using element dopants [9]. Several groups have reported that modification of TiO_2_ with metal/non-metal ion such as N, Cr, Pt, Nb, Co, and polyaniline [8-17] could adjust energy band to optimum values and thus high conductivity paths may be achieved. Furthermore, some theoretical calculations have been also performed to suggest that metal/non-metal ion doping in TiO_2_ could have significant impact on the bandgap alteration.

TiO_2_ doped with certain amount of Ni has been reported. Yao et al. reported that the substitution of Ti^4+^ ions in the anatase or rutile TiO_2_ lattice with a certain amount of Ni^2+^ could expand the optical absorption range by changing bandgaps [18]. Wisitsoraat et al. reported that TiO_2_ thin films doped with 0 to 10 wt.% content NiO_*x*_ could have a gas-sensing capability for ethanol, acetone, and CO at 300°C [19]. Nakhate et al. used hydrothermal method to prepare Ni-TiO_2_ film and studied the effect of Ni doping concentration on the photoactivity for methylene blue degradation [20]. Patil et al. found that the nanostructured 2.5% Ni-doped TiO_2_ thin film was very sensitive to liquified petroleum gas at 250°C [21]. Park et al. reported that electronic structure of Ni-doped TiO_2_ oxide could have a paramagnetic ground state and Chen et al. explored the ferromagnetic mechanism of Ni-doped TiO_2_ by series of density functional calculations [22,23].

To date, rare works have been reported on the hydrogen sensing properties of Ni-doped TiO_2_ oxides except for our recent work on the fabrication of Ni-doped TiO_2_ nanotubes and demonstration of the nanotubes' hydrogen sensing capability at elevated temperatures [24]. Furthermore, there is no theoretical investigation on hydrogen adsorption in Ni-doped TiO_2_ oxide. In the present work, Ni-doped TiO_2_ nanotubes annealed at 525°C were fabricated for hydrogen sensing testings at both room temperature and elevated temperatures. In addition, a first-principles study on the surface adsorption models of the Ni-doped TiO_2_ oxide was also carried out to for a better understanding of the good hydrogen sensing capability of the Ni-doped oxide.

## Methods

### Materials and film fabrication

Equiatomic NiTi (nominal composition 50.8 at.% Ni) plates with a size of 15 mm × 10 mm × 1 mm were first ground and polished with #2000 SiC emery papers and then ultrasonically cleaned with absolute alcohol. Finally, they were rinsed with deionized water and further dried in a nitrogen stream. Electrochemical anodization at 30 V was carried out with a non-aqueous electrolyte of 5% ethylene glycol/glycerol containing 0.30 M (NH_4_)_2_SO_4_ and 0.4 M NH_4_F. The anodization was conducted for 90 min. The as-anodized samples were rinsed in sequence with ethanol and deionized water and dried in an air stream. They were then annealed at 525°C for 1 h in air to obtain crystallized nanotubes. Circular Pt electrodes with a thickness of 200 nm were deposited onto surfaces of the crystallized nanotube samples through sputtering. Conductive wires were connected to the Pt electrode with conductive paste. The nanotube samples (with corresponding alloy substrate) were put in a ceramic boat for further sensing test.

### Characterization of nanostructure films

The phase structures of the as-annealed samples were characterized by X-ray diffraction (XRD, D/max 2,550 V). Grazing incident diffraction (GID) with an incident angle of 1° was carried out during the XRD testing. The surface morphologies of the as-anodized and as-annealed nanotube samples were examined using a scanning electron microscope (SEM; FEI SIRION 200, FEI Company, Hillsboro, OR, USA) equipped with energy dispersive X-ray (EDX; Oxford INCA, Oxford Instruments, Abingdon, Oxfordshire, UK). Surface compositions and composition distribution along the depth of the Ni-doped nanotubes were characterized with X-ray photoelectron spectroscopy (XPS; ESCALAB 250, Thermo Fisher Scientific, Hudson, NH, USA).

### Analytical determinations of hydrogen sensors

**Figure 1:**
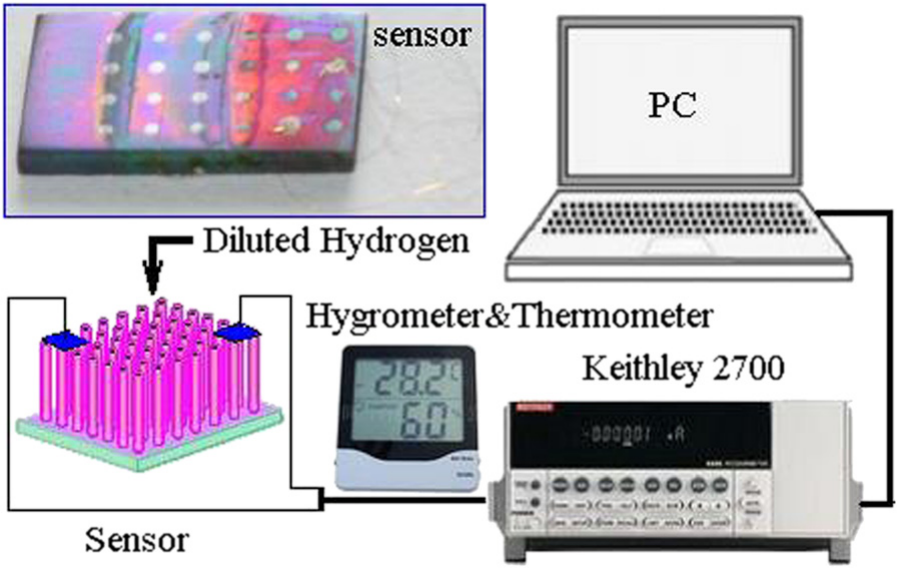
**Schematic diagram of the Ni-TiO**_**2 **_**nanotube sensor and testing system.**

A Keithley 2700 multimeter (Keithley Instruments Inc., Cleveland, OH, USA) was used to record the resistance variation of the nanotube sensor in the N_2_ atmospheres containing a certain concentration of hydrogen. All of the hydrogen sensing testings were carried out under laboratory condition with a room temperature of 25°C and constant humidity at 45% in air. Since N_2_ background atmosphere could not support a repeatable sensing response of the oxide sensor, we directly use the environmental air as the recovering atmosphere. The sensor was put on a hot plate, and the testing atmosphere was fed directly toward the sensing surface. The gas feeding tube had an outer diameter of 8 mm and the distance between the tube mouth and the sensor was 10 mm. The total flow rate of the testing atmosphere was 1 L/min. Sensor response in this paper is defined as *S* = (*R* − *R*_0_) / *R*_0_, in which *R* and *R*_0_ represent the resistance of the sensor in air and tested gas, respectively. A schematic diagram of the sensor structure and testing system is shown in Figure 1.

## Results

**Figure 2:**
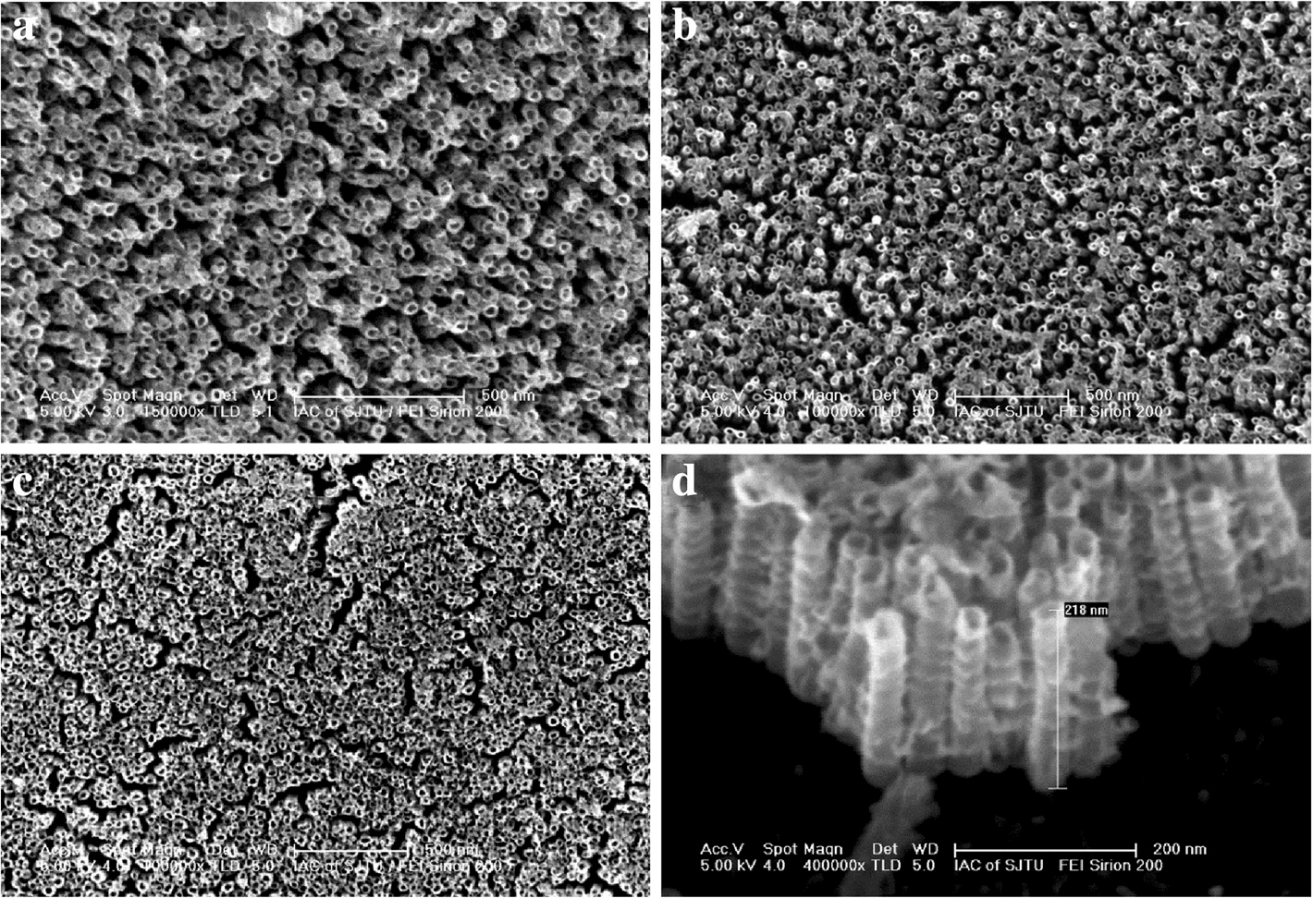
**SEM images of Ni-doped TiO**_**2 **_**nanotubes. (a)** After anodization. **(b)** Annealed at 525°C. **(c)** Annealed at 625°C. **(d)** Cross-section image of the nanotubes annealed at 525°C.

After anodization, the samples were characterized using SEM and EDX. Figure 2 presents the surface morphology of the nanotube arrays after anodic oxidation process and annealing at elevated temperatures. The anodization process resulted in the formation of aligned nanotubes with a diameter of about 30 nm and length of about 220 nm (Figure 2a,d). In comparison with the as-anodized nanotubes (Figure 2a), the tubular surface morphology of the oxide nanotubes annealed at 525°C did not change apparently (Figure 2b). This suggests that the nanotube arrays could bear such a high temperature. With an increase of the heat treatment temperature to 625°C, the top ends of nanotubes became slightly collapsed (Figure 2c), although they could still keep their tubular structures.

**Figure 3:**
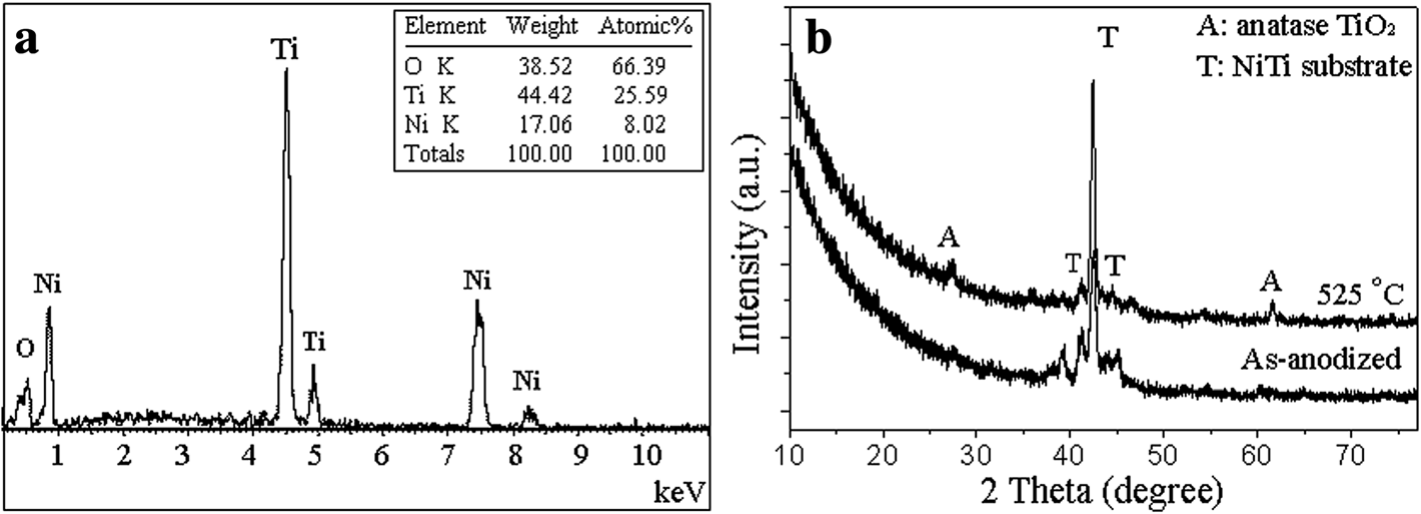
**The phase structure and composition analysis of the Ni-doped TiO**_**2 **_**nanotubes. (a)** EDX analysis pattern of the as-annealed samples. **(b)** XRD pattern of the as-anodized samples and as-annealed samples.

Figure 3a presents EDX analysis of the middle part of the nanotubes annealed at 525°C. It indicates that the nanotubes consisted of three elements, i.e., Ni, Ti, and O. The atomic percentage of the Ni, Ti, and O elements in the nanotubes was 7.92%, 24.29%, and 67.79%, respectively. Figure 3b presents XRD pattern of the Ni-doped TiO_2_ annealed at 525°C. The 2*θ* of 26° and 42° corresponded to the diffraction peak of Ni-doped anatase TiO_2_ phase, indicating that the heat treatment at 525°C resulted in a crystallization of the amorphous nanostructures to anatase phase. The strong diffraction peaks corresponding to NiTi and Ni were also found due to the formation of thin layer (several hundreds of nanometers in thickness) of the nanotube array on alloy substrate. In comparison with undoped TiO_2_ nanotubes [5,7], the Ni-doped nanotubes almost had the same crystallization temperature, which suggests that the doping of Ni element did not significantly affect the amorphous-to-anatase phase transformation of the anodic oxide [25].

**Figure 4:**
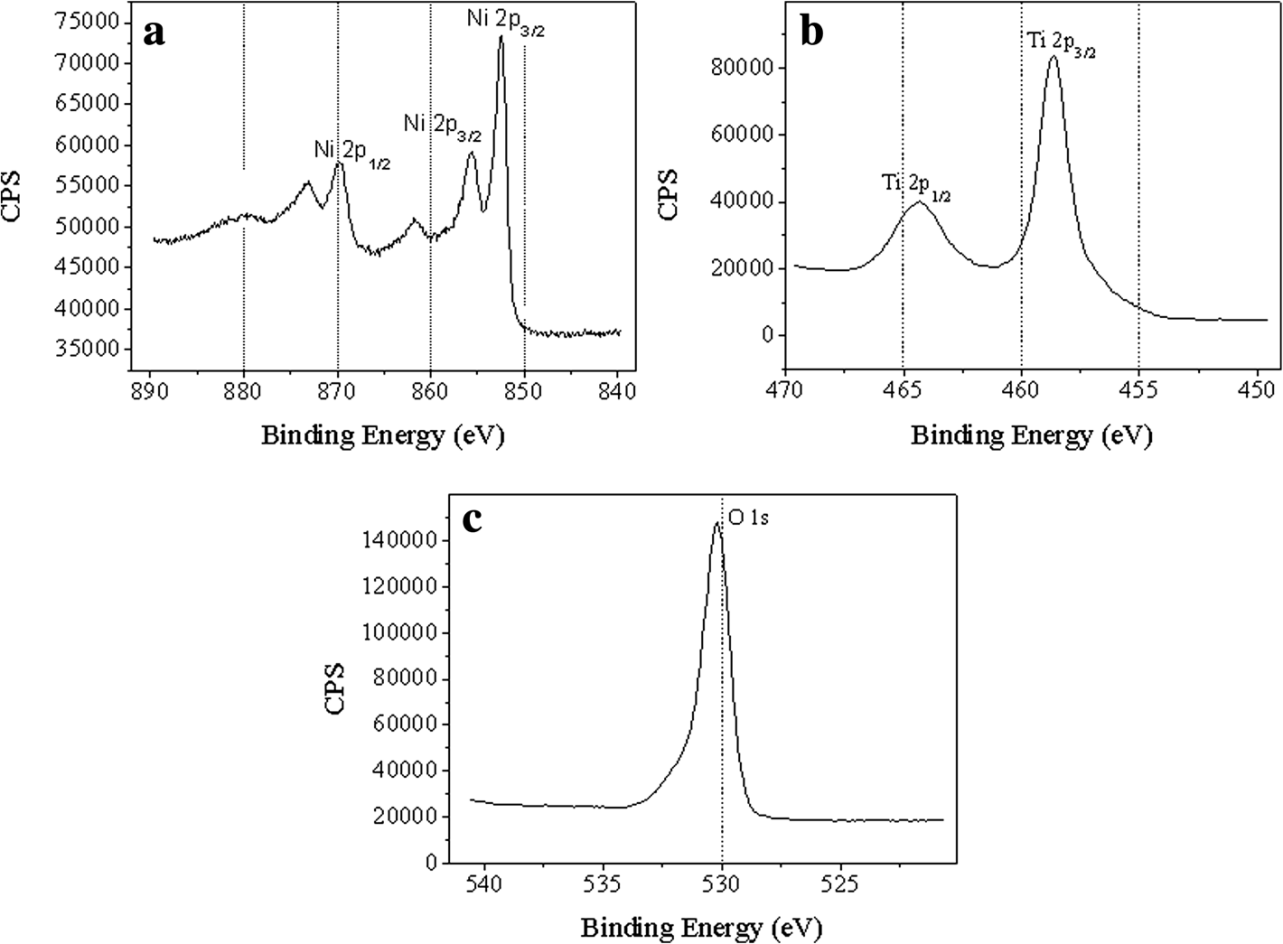
**XPS analysis of the Ni-doped nanotubes. (a)** Ni 2*p* spectrum **(b)** Ti 2*p* spectrum, and **(c)** O 1 *s* spectrum.

Figure 4 presents XPS spectrum of the Ni-TiO_2_ nanotubes annealed at 525°C. The oxide nanotubes consisted of Ti, O, and Ni elements, which is in agreement with our EDX analysis. The 2*p*_3/2_ and 2*p*_1/2_ peaks in the Ti 2*p* spectrum (Figure 4a) are characteristic of titanium oxide. The peaks located at 458.6 and 464.4 eV are assigned to Ti 2*p* electrons of Ti^4+^ ions [18,25]. However, no peak corresponding to metallic state (Ti^0^) was detected. As widely reported in literatures, the most intense Ni 2*p*_3/2_ and Ni 2*p*_1/2_ peaks (located at about 852.4 and 869.7 eV, respectively) are characteristic of metallic Ni [26,27], which should be mainly attributed to the alloy substrate. In the Ni 2*p*_3/2_ region, the peak at 855.6 eV could be attributed to Ni^2+^ ions in an oxygen-containing environment [27]. This reveals that Ni was doped in the TiO_2_ lattice. The Ni dopant mainly existed as Ni^2+^ and bonded with O^2−^[28].

Longitudinal composition of Ni-Ti-O oxide nanotubes was characterized by XPS with an etching depth up to 20 nm. The atomic percentage of the Ni, Ti, and O elements was 7.11%, 24.12%, and 62.97%, respectively. It could be found that the chemical compositions of the three elements only slightly varied with our EDX results. The atomic ratio of Ni and Ti elements was much lower than the original atomic ratio of the NiTi alloy substrate. Obviously, during the anodization process, Ni element could be easily corroded in the electrolyte solution.

**Figure 5:**
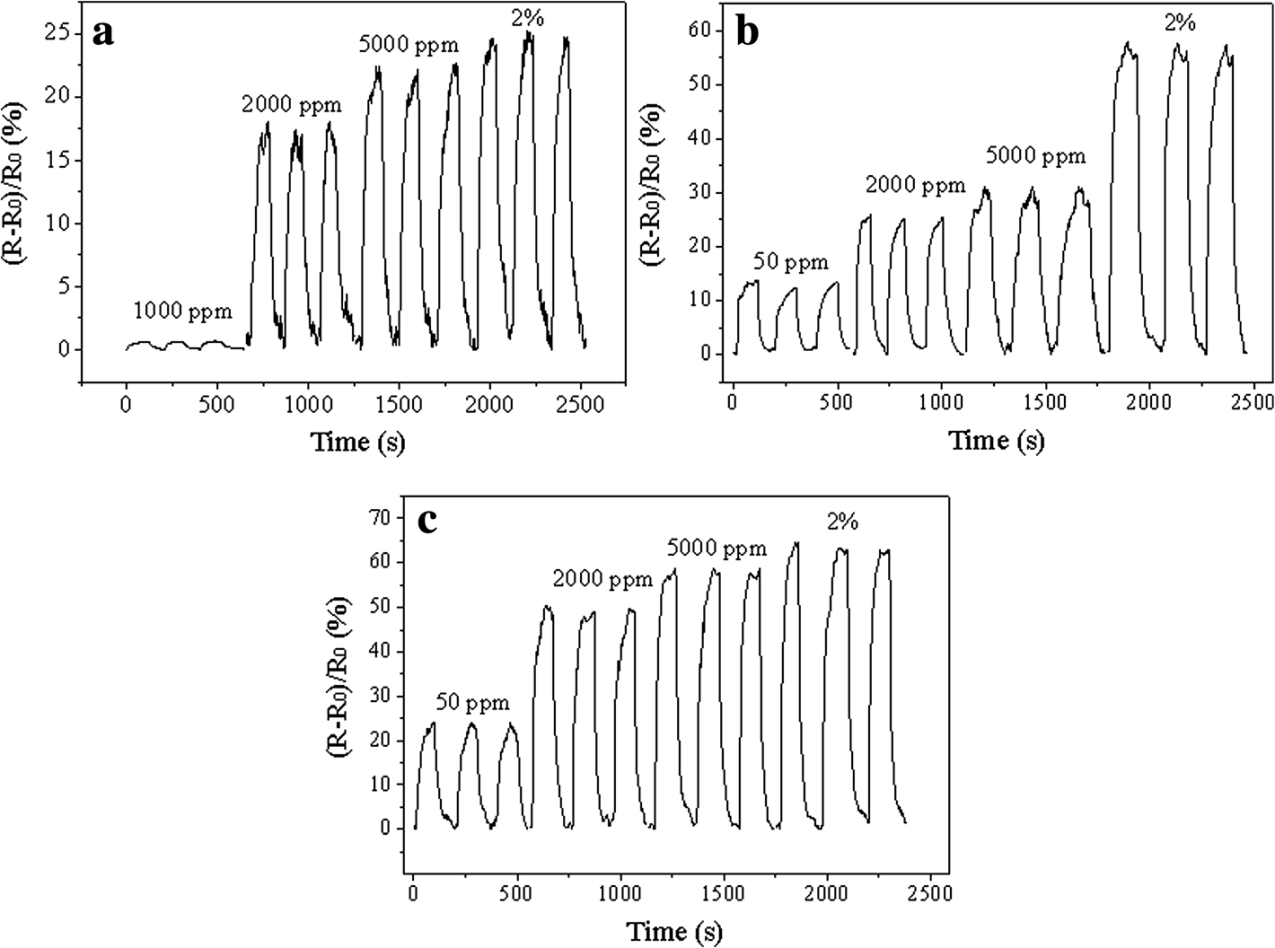
**Response curves of the Ni-doped TiO**_**2 **_**sensor exposed to various hydrogen concentrations. (a)** 25°C **(b)** 100°C, and **(c)** 200°C.

Figure 5 shows the saturation or maximal response of the Ni-doped nanotube sensors to the hydrogen-containing atmosphere and air background at operating temperatures of 25°C, 100°C, and 200°C. In comparison with the previously reported Ni-doped TiO_2_ nanotubes annealed at 425°C [24], the nanotubes annealed at 525°C here could have a better hydrogen sensing capability by showing an enhanced response and there were no baseline drift phenomena. At 25°C, the nanotube sensor could only detect the atmosphere with more than 1,000 ppm hydrogen. The response increased with increase of hydrogen concentration (Figure 5a). In response to the 1,000 and 2,000 ppm dilute hydrogen atmospheres, 0.6% and 17% changes in resistance could be found, respectively. In addition, for the 2% hydrogen atmosphere, a 25% change in resistance and response time (defined as the time taken for the sensor's resistance to reach the 90% of the steady-state resistance) of 80 s could be found. The sensor could detect as low as 50 ppm hydrogen atmosphere at 100°C and 200°C (Figure 5b,c), which demonstrated a much better sensing capability than the nanotubes annealed at lower temperature.

**Figure 6:**
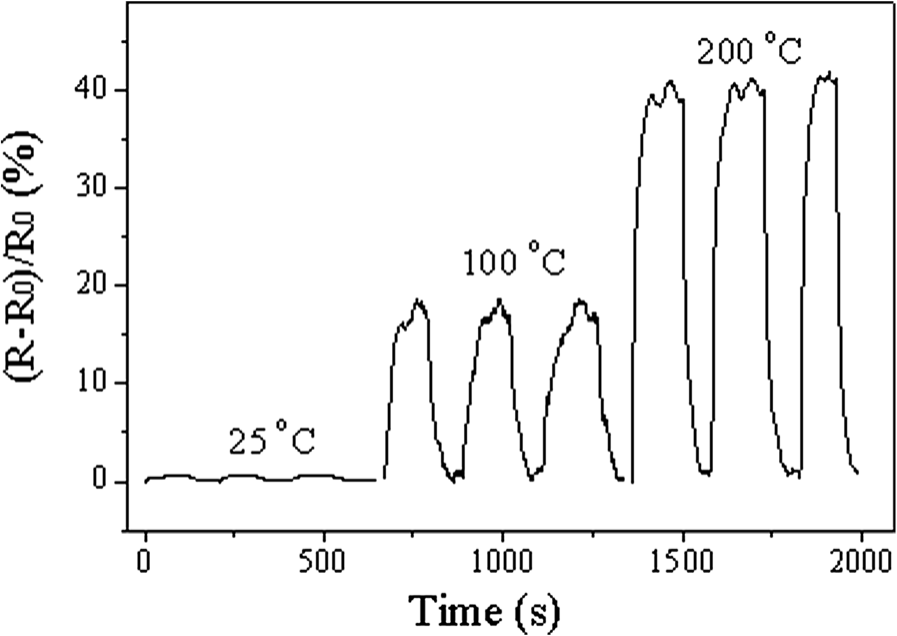
**Response curves of Ni-doped TiO**_**2 **_**sensor exposed to 1,000 ppm dilute hydrogen atmosphere at different temperatures.**

Figure 6 shows a temperature-dependent sensing of the Ni-doped TiO_2_ sensor to 1,000 ppm hydrogen atmospheres. It also found that the sensor response speed increases with the increase of temperatures.

At 25°C, only 0.6% change in resistance could be found for the 1,000 ppm H_2_ atmosphere. The response was much smaller than the response at 100°C and 200°C. A 14% change in resistance could be found at 100°C, and a 40% change in resistance at 200°C was found. Obviously, the nanotube sensor had a remarkable performance by showing a wide detection range and a quick response/recovery at elevated temperatures.

## Discussion

To explore the effect of Ni doping on the bandgap and interaction of hydrogen with TiO_2_ oxide, the first-principles calculations were performed with the CASTEP code [29] based on density functional theory. According to Mitsui et al., hydrogen molecule adsorbed on the Pt catalyst could separate into hydrogen atoms and then diffuse to oxide nanotube [30]. The hydrogen sensing performance of TiO_2_-based nanotubes is dependent on the formation of a surface electron accumulation layer induced by the chemisorption of hydrogen atoms on the nanotube surface. Many research has shown that anatase TiO_2_ (101) is the most stable and frequently exposed surface of anatase oxide, and the diffusion of hydrogen atoms in anatase TiO_2_ (101) is much easier than in other crystal planes [31-33]. Therefore, in the present study, we investigate the interaction of hydrogen atom with Ni-doped anatase TiO_2_ (101) surface.

Based on the our XPS results, the adsorption of atomic hydrogen was simulated by placing the H atom on different surface sites in a (1 × 1) unit cell (which includes three titanium atoms, one nickel atom, and eight oxygen atoms) modeling high hydrogen coverage [31]. Spin-polarized DFT calculations were performed within the generalized gradient approximation (GGA) and the periodic plane wave approach, using the Perdew-Burke-Ernzerhof (PBE) exchange-correlation functional and ultrasoft pseudopotentials [34-36]. Bulk anatase TiO_2_ belongs to the tetragonal $D_{4h}^{19}/\mathrm{amd}$ space group with lattice parameters of *a* = 3.782 Å and *c* = 9.502 Å [37]. We applied the projector-augmented wave method with 3 × 3 × 1 k-point grids and cut-off energy of 380 eV, which ensures an energy convergence to within 1 to 2 meV/atom. To simulate surfaces, a vacuum region of 15 Å was embedded along the surface normal to eliminate the unwanted interaction between the slab and its period images. In different geometry optimizations of two-dimensional periodic slab, the lattice constants were fixed at these values, while the positions of all of the Ti, Ni, and O atoms were allowed to vary.

**Figure 7:**
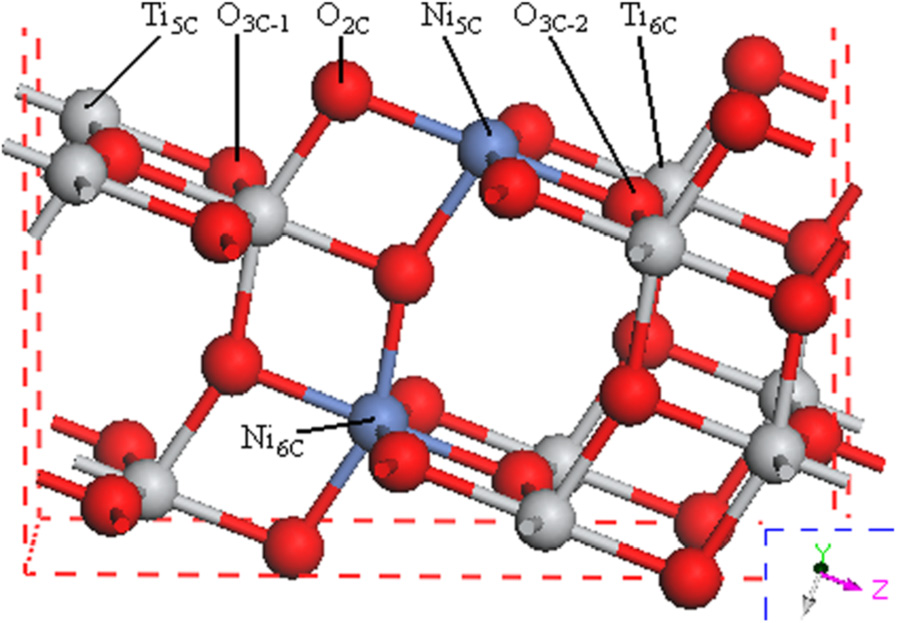
**Atomic geometries of Ni-doped TiO**_**2 **_**(101) surface**. The gray, red and blue balls represent O, Ti, and Ni atoms, respectively. O_2C_, O_3C_, Ti_5C_, Ti_6C_, Ni_5C_, and Ni_6C_ mean the most probable sites for the adsorption.

Figure 7 shows the surface sites model. The O fills either twofold (O_2C_) or threefold (O_3C-1_ and O_3C-2_) coordinated sites. The Ti fills fivefold (Ti_5C_) or sixfold (Ti_6C_) coordinated sites, and the Ni fills fivefold (Ni_5C_) or sixfold (Ni_6C_) coordinated sites. As for the H adsorption, seven candidate models [32] had been taken into account with the H adsorbed on Ti_5C_, Ti_6C_, Ni_5C_, Ni_6C_, O_2C_, and O_3C-1_, and O3_C-2_ sites. To determine the most energetically favorable model, adsorption energy (*E*_ads_), which is defined as reversible energy needed to separate an adsorption system into a Ni-doped surface (*E*_surf_) and free hydrogen (*E*_H_), is calculated using the following equation:

(1) $$\Delta E_{\mathrm{ads}}=E_{H+\mathrm{surf}}-E_{\mathrm{surf}}-E_{H}.$$

where *E*_surf_ is the energy of the Ni-doped TiO_2_ (101) surface, *E*_H + surf_ is the total energy of the adsorption model, and *E*_H_ is the energy of dissociated atomic hydrogen (−12.62 eV).

**Figure 8:**
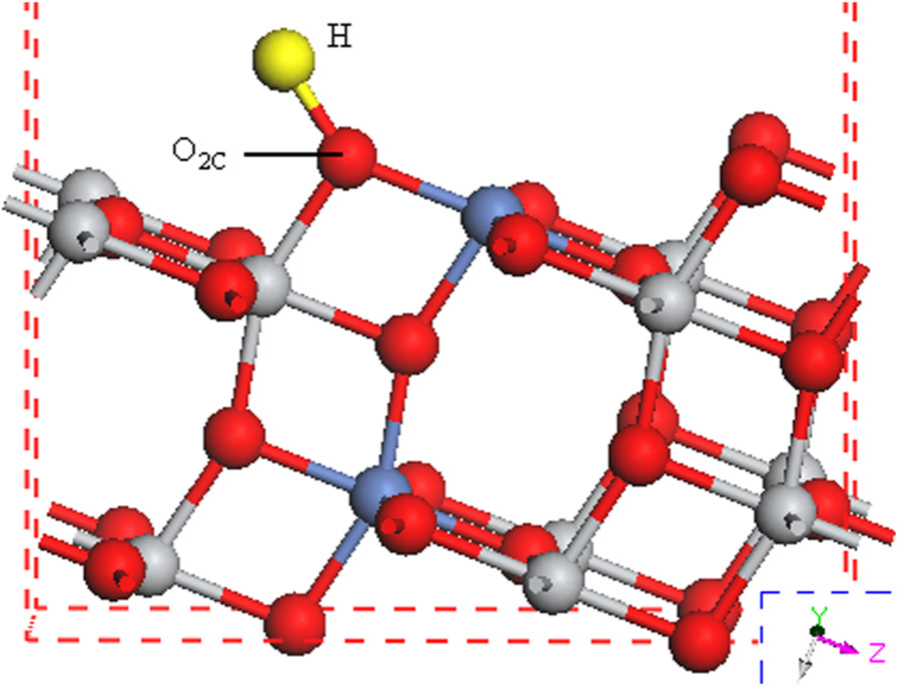
**Calculated structures of Ni-doped TiO**_**2 **_**(101) surface adsorption of one dissociated hydrogen atom at O**_**2C **_**site.**

The adsorption energies of the above seven calculated models for H adsorption on Ni-doped TiO_2_ (101) surface are shown in Table 1. Theoretically dissociated atomic hydrogen impinging on the surface from the gas phase could adsorb to any of the exposed oxygen, nickel, or titanium sites. But our simulation results indicate that in comparison with the other sites, atomic hydrogen could easily stick to the surface oxygen atoms particularly at O_2C_ site, which is consistent with results from Islam et al. [31]. This is because the structural deformation induced by H adsorption at O_2C_ site is more evident in comparison with the two-dimensional solid surface of Ni-doped TiO_2_ (101) [31]. A preferred model of the hydrogen adsorption on O_2C_ site of Ni-doped (101) surface is shown in Figure 8.

**Table 1:** Hydrogen adsorption energy for different models

**Ni-TiO**_**2**_	**O**_**2C**_	**O**_**3C-1**_	**O**_**3C-2**_	**Ni**_**5C**_	**Ni**_**6C**_	**Ti**_**5C**_	**Ti**_**6C**_
Δ*E*_ads_	−4.73	−3.48	−1.22	−1.77	−3.87	−3.88	−3.88

**Figure 9:**
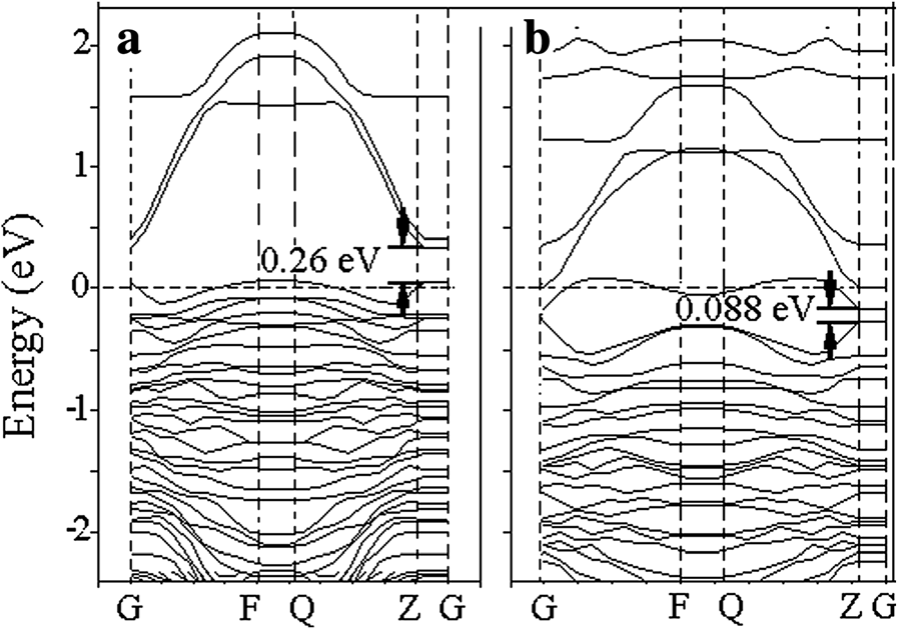
**Band structure of the (101) surface. (a)** Before and **(b)** after hydrogen adsorption at Ni-doped TiO_2_ surfaces. The Fermi level is set to 0.

To investigate the doping effect of Ni on the electronic structures, the band structure was calculated by replacing one Ti site of TiO_2_ lattice with a Ni atom (Figure 9a). The energy zero refers to the Fermi level. Compared to the bandgap value of 2.40 eV for undoped TiO_2_[22,23], the remarkable feature in the energy band for the Ni-doped TiO_2_ was that the bandgap greatly decreased to 0.26 eV. This implies that high-concentration Ni doping in anatase TiO_2_ could result in a narrow bandgap and reveal metallic characteristic in comparison with the low-concentration doping system [18,19]. Therefore, the electron transfer rate in the Ni-doped oxide could be greatly enhanced and electrons could be easily excited from valence band to conduction band [38,39], which enabled better conductivity when the oxide was exposed to the hydrogen-containing atmospheres. This may be a major reason for our nanotube sensor to have improved hydrogen sensing properties at room temperature. In our work, we use Pt as the electrodes to dissociate hydrogen molecular and use a higher crystallization temperature to obtain more anatase phases. The Pt electrode and the increase of anatase phase should have also enhanced the hydrogen sensing behavior of the Ni-doped TiO_2_[5,7].

As shown in Figure 9a, the relative position of the Fermi level of Ni-doped TiO_2_ dramatically shifts down closer to the valance band maximum. This directly proves that the highly doped TiO_2_ is a p-type semiconductor [40]. These characteristics are consistent with the previous reports by Wisitsoraat and Ganesh as well as Wang et al. [19,41,42]. Generally, when a p-type semiconductor was exposed to reducing gases such as CO and H_2_, the reducing gases donated electrons to the valence band by reducing the number of holes and thus increasing the electrical resistance [37,43-45]. In our experiment, the resistance of the Ni-doped TiO_2_ nanotubes increased after exposure to hydrogen-containing atmosphere. This result well accords with our simulation result.

After H adsorption, the bandgap of the Ni-doped TiO_2_ further decreased to 0.088 eV (Figure 9b). It indicates that hydrogen atom could easily adsorbed in the Ni-doped TiO_2_ oxide to result in a smaller bandgap. This means that Ni doping could make the hydrogen adsorption easier in terms of energy. The ease of hydrogen adsorption in the Ni-doped TiO_2_ system would naturally lead to enhanced hydrogen sensing behavior.

**Figure 10:**
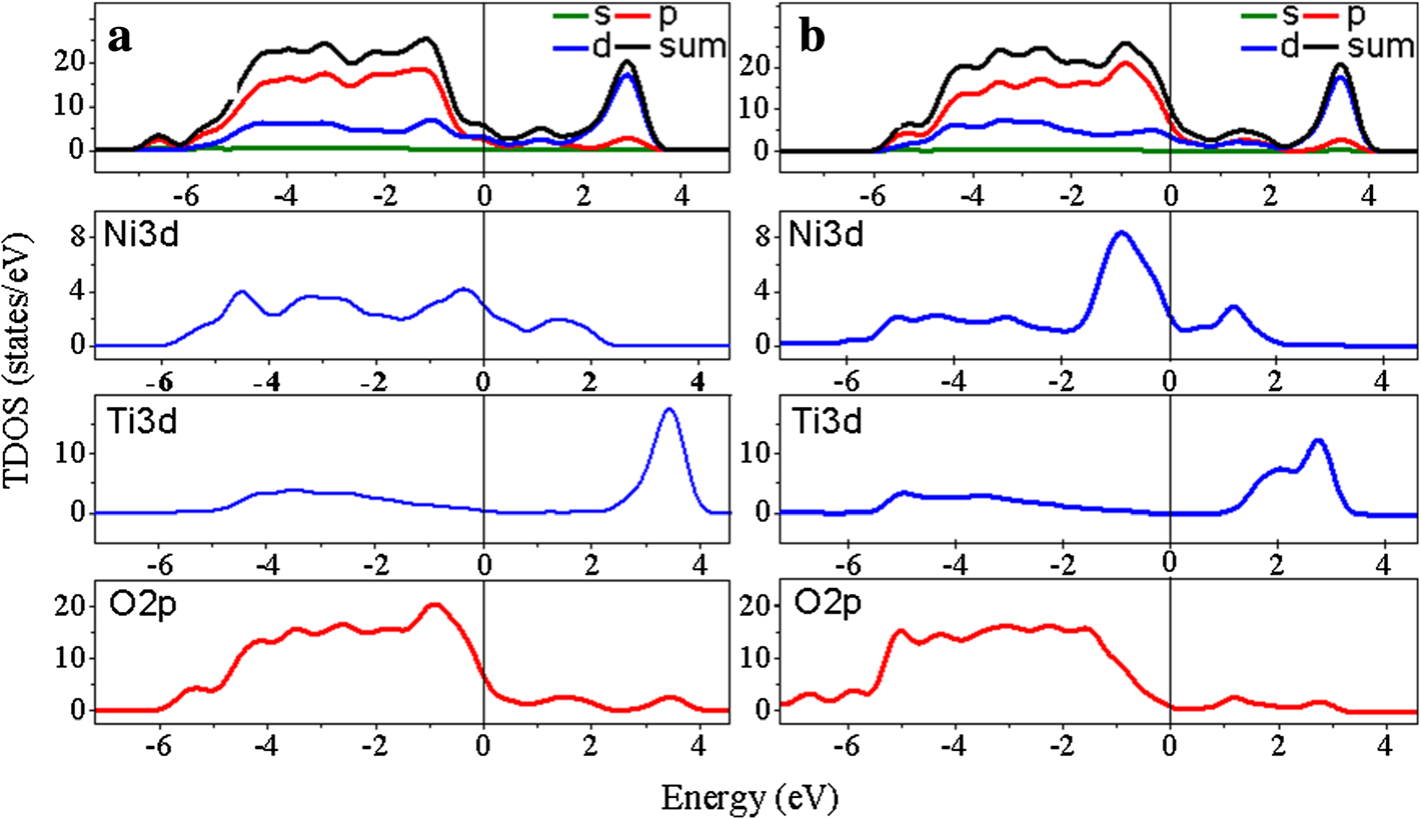
**Total and partial DOS of the (101) surface. (a)** Before and **(b)** after hydrogen adsorption at Ni-doped TiO_2_ surfaces.

The total density of states (TDOS) and partial density of states (PDOS) of the Ni-doped TiO_2_ oxide were calculated. Figure 10 shows DOS of the Ni-doped TiO_2_ (101) surface before and after hydrogen adsorption. The energy zero refers to the Fermi level. Adsorption of hydrogen resulted in a significant change of band structure. The Fermi level shifted towards low-energy direction. The Ni 3*d* states at the top of the valence band were greatly enhanced, and Ti 3*d* states at the bottom of the conduction band were slightly weakened.

It can be found that the top of the valence bands were dominated by the O 2*p* orbital, the bottom of conduction bands was dominated by the Ti 3*d* orbital, and the impurity band was dominated by the Ni 3*d* orbital [46]. In comparison with undoped anatase TiO_2_[10,45], one important feature of the Ni-doped system is that this oxide had an acceptor impurity level (Ni^2+^ inside TiO_2_ lattice) and half-metallic state formation before and after adsorption. As a 3*d* transition metal, Ni has more valence electrons than those of Ti and Ni dopants. Thus, it creates defect states in the bandgap, leading to an impurity level. In undoped or pure TiO_2_, the electrons usually cannot be easily excited from the valence band to the conduction band until a sufficient amount of energy is available. In Ni-doped TiO_2_, impurity level generated in the bandgap could give a migration pathway for the hydrogen to overcome activation barrier [30]. As a result, more electrons could be elevated to conduction band and thus lead to a higher sensitivity [37,46,47].

In our experiment, we found that the hydrogen sensing properties of the Ni-doped TiO_2_ nanotubes were enhanced with increase of the working temperature. This is because an increased operating temperature could accelerate the diffusivity of the hydrogen atoms into the nanotubes and thus lead to a higher sensitivity [48]. The Ni-doped TiO_2_ oxide was theoretically found to have favorable hydrogen-oxide interaction compared to pure TiO_2_ oxide. Our simulation results may shed light on better understanding of gas-sensing behaviors of various kinds of doped TiO_2_ oxides.

## Conclusions

Ni-doped titania nanotubes with anatase-phase structures were fabricated through anodization and annealing at 525°C. The doped nanotubes were found to be sensitive to hydrogen atmospheres in the temperature range from room temperature to 200°C. A wide-range sensing of 50 ppm to 2% H_2_ with the robust nanotube sensor was realized. First-principles simulation of the electronic properties and hydrogen sensing behavior revealed that Ni doping played an important role in improving the hydrogen response of anatase TiO_2_ by narrowing the bandgap, the mechanism of which was clarified with theoretical surface models. The simulation results verified the change of the semiconductor characteristic and resistance before and after hydrogen interaction.

## Competing interests

The authors declare that they have no competing interests.

## Authors' contributions

ZL participated in the experiment design, carried out the experiments, tested the thin films, and wrote the manuscript. DD, LQ, CN, and WX designed the experiments and testing methods and helped to proofread the manuscript. All authors read and approved the final manuscript.
